# Control of fibrotic changes through the synergistic effects of anti-fibronectin antibody and an RGDS-tagged form of the same antibody

**DOI:** 10.1038/srep30872

**Published:** 2016-08-03

**Authors:** Anil Tiwari, Rajendra Kumar, Jagat Ram, Maryada Sharma, Manni Luthra-Guptasarma

**Affiliations:** 1 Department of Immunopathology, Postgraduate Institute of Medical Education and Research, Chandigarh, 160012, India; 2 UGC Centre of Excellence in Applications of Nanomaterials, Nano particles and Nanocomposites, Panjab University Chandigarh, 160014, India; 3 Department of Ophthalmology, Postgraduate Institute of Medical Education and Research, Chandigarh, 160012, India.

## Abstract

TGF-β and myofibroblasts play a key role in fibrosis, characterized by aberrant synthesis and deposition of extracellular matrix (ECM) proteins, such as fibronectin (Fn) and collagen type I. There are two major roles played by integrins in the fibrotic pathology: (i) Fn-integrin interaction, coupled with cytokines like TGF-β, facilitates the self-polymerization of Fn and regulates cell–matrix fibrillar adhesions, thereby promoting fibrillogenesis; (ii) Integrin interaction with an RGD (arginine-glycine–aspartic) consensus sequence in the latent TGF-β, resulting in its activation. This study describes an anti-fibrotic strategy using a combination of two antibodies: Fn52 (targeted against the N-terminal 30 kDa region of fibronectin, a major site for Fn self-association), and its engineered form, Fn52RGDS (which binds to integrins). Interestingly, a synergistic effect of the cocktail in causing a decline in fibrotic features was confirmed in the context of fibrotic posterior capsular opacification (PCO), mediated by the lens epithelial cells (left behind after cataract surgery). Inclusion of Fn52RGDS to Fn52 aids in better diffusion of the antibodies; such combination therapies could be useful in the context of pathologies involving extensive remodeling of the fibronectin matrix, where the thick ECM offers a major challenge for efficient drug delivery.

Extracellular matrix (ECM) is a dense meshwork of proteins and plays a pivotal role in regulating cell proliferation, differentiation, cell survival and migration^1^. The adhesion of cells to the ECM is mediated through interaction with adhesion proteins like fibronectin (Fn), laminin, collagen and others. Unlike other ECM components which are capable of self polymerization^2^, the polymerization of fibronectin is dependent upon its interaction with cell surface receptors (integrins), for exposure of critical cryptic sites necessary for interaction with other fibronectin molecules and polymerization^3^,^4^,^5^.

Fibronectin is present in two isoforms: the soluble fibronectin, synthesized mainly by liver hepatocytes, is found in the circulating blood plasma, and interacts with cell surface receptors to assemble into fibrillar matrix leading to the deposition of insoluble fibronectin. The insoluble form of fibronectin, is also referred to as cellular fibronectin, and is synthesized by many cell types, including chondrocytes, synovial cells, endothelial cells, fibroblasts, and myocytes^4^.

Fibronectin matrix assembly is a dynamic and continuous event, and a continuity in fibronectin polymerization is essential for maintaining the matrix composition and stability^6^. The polymerization of fibronectin is initiated by the binding of FnI_1–5_ within the 70-kDa domain of Fn to cell-surface integrin receptors, followed by binding of FnIII_9–10_ to α5β1 integrin^7^, which activates the integrins. This leads to induction of cytoskeletal reorganization of the actin cytoskeleton and contractility of the cells, causing unfolding of the tethered fibronectin molecules, which exposes cryptic binding sites of fibronectin that are important for Fn-Fn intermolecular interactions, which result in Fn polymerization^8^.

Excessive deposition of Fn which precedes collagen deposition, is a characteristic feature of fibrosis^9^. Posterior capsular pacification (PCO) is one such fibrotic disorder, caused by lens epithelial cells (LECs) retained in the capsular bag following cataract surgery. These cells respond by undergoing a wound healing response including cell proliferation, migration and transformation into myofibroblasts, along with excessive synthesis and deposition of ECM components such as fibronectin^10^.

We have previously shown in the context of a fibrotic disorder, proliferative vitreoretionopathy (or PVR), that scFv antibodies - Fn52 (selected by phage display technology against the N-terminal 30 kDa region of fibronectin), and its engineered form Fn52RGDS (which also binds to cell surface integrins) are effective in downregulating some of the important features of this fibrotic pathology, including cell migration, fibronectin deposition and collagen gel contraction^11^. The present study highlights that simultaneous disruption of the fibronectin matrix by inhibiting fibronectin polymerization, together with inhibition of Fn-integrin interactions, by using a cocktail of two antibodies, could be potentially significant in preventing fibrotic pathologies like PCO.

## Results

### Effect of the scFv antibodies on cell viability and proliferation

Viability of lens epithelial cells (LECs) was evaluated by MTT assay. scFv antibodies Fn52, Fn52RGDS and a combination of both Fn52 and Fn52RGDS were used in a dose dependent manner from 10 μg/ml to 100 μg/ml. Addition of scFv antibodies (alone or in combination) did not result in any significant decrease in viability of lens epithelial cells (Supplementary Fig. 1A).

To assess the effect on proliferation, the extent of BrdU intake was determined in the lens epithelial cells by cell ELISA. scFv Fn52 or Fn52RGDS alone, at a concentration of 100 μg/ml, caused significant inhibition of epithelial cell proliferation, while the combination of Fn52 and Fn52RGDS, showed the same extent of inhibition at a concentration starting from 50 μg/ml. scFv O52 (irrelevant negative control) did not show any effect on proliferation at any concentration (Supplementary Fig. 1B). Control indicates the condition where cells were seeded in the absence of scFv antibody. Accordingly, for the rest of the experiments, a concentration of 50 μg/ml was used for Fn52, Fn52RGDS and O52. When the scFvs were used in combination, Fn52 and Fn52RGDS were used at a concentration of 25 μg/ml each, unless stated otherwise.

### scFv antibodies directed against fibronectin block fibronectin polymerization and alter actin-fiber rearrangement

**Figure 1:**
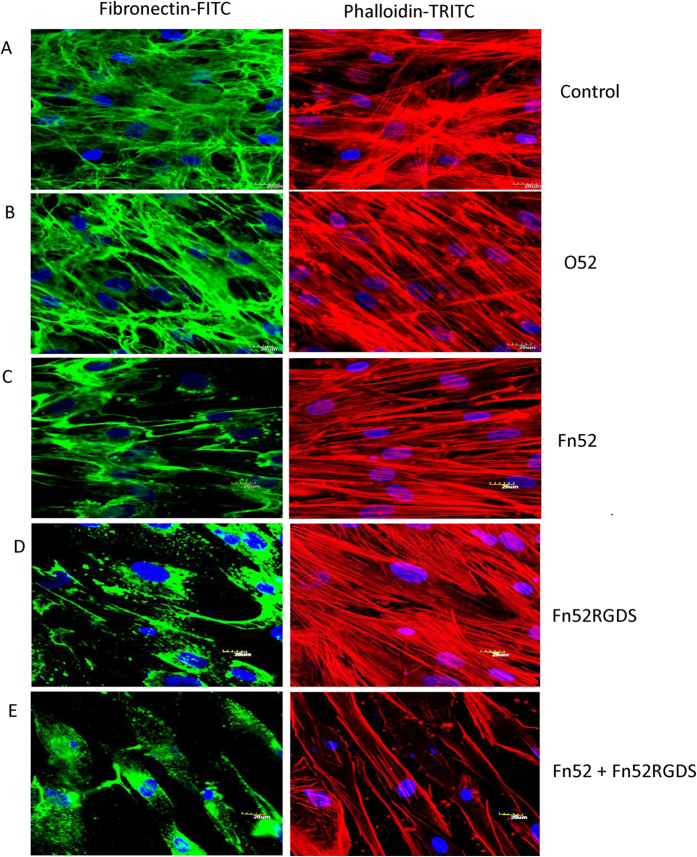
Evaluation of fibronectin matrix deposition and F-actin stress fiber rearrangement by immunofluorescence. Anti-fibronectin antibody was used for imaging fibronectin matrix deposition (green) and TRITC-Phalloidin (red) for F-actin stress fiber arrangement. (**A**) absence (control) of scFv antibody; (**B**) O52 (irrelevant control antibody); (**C**) Fn52 (50 μg/mL); (**D**) Fn52RGDS (50 μg/mL); and (**E**) combination of Fn52 and Fn52RGDS (25 ug/ml each). All these experiments were done in the presence of TGF-β2 (2 ng/ml). Images were acquired at 60X magnification; scale bars represent 20 μm.

Lens epithelial cells were grown along with scFv antibodies (50 μg/ml) and TGF-β2 for evaluating the effect on fibronectin polymerization and actin-stress fiber rearrangement. In each case, there was reduction in the extent of fibronectin polymerization as compared to the two controls, i.e., in the presence of an irrelevant antibody (scFv O52), or the absence of any scFv antibody (Fig. 1 Panels A–E). Dense and continuous fibrils of fibronectin were seen in the control cultures; in the case of scFv Fn52, fibronectin fibrils could be observed to be associated with the cell surface, suggesting that there was no effect on cell-fibronectin interaction. The pattern of fibronectin assembly in the presence of scFv Fn52RGDS was distinct from scFv Fn52; there were no continuous fibronectin fibrils, and only a punctate pattern of fibronectin deposition could be observed, suggesting the absence of cell-fibronectin interaction. The status of fibronectin polymerization in the presence of the combination of the two antibodies (Fn 52 and Fn 52RGDS) is shown in Fig. 1 Panel E, showing a dramatic loss of fibronectin matrix. Next, we evaluated the actin stress fiber formation in these cultures. As seen in Fig. 1, there was no appreciable rearrangement of actin-stress fibers in the presence of either Fn52 or Fn52 RGDS when compared to the control (Panels A–D). However, the combination of Fn52 and Fn52RGDS, led to a striking alteration in the actin fiber arrangement; there was a drastic decrease in actin-stress fiber formation, along with altered localization of these fibers (towards the cell periphery), as compared to the control where extensive actin-stress fibers distributed throughout the cell were observed (Fig. 1E).

### Evaluation of LEC migration and MMP expression

**Figure 2:**
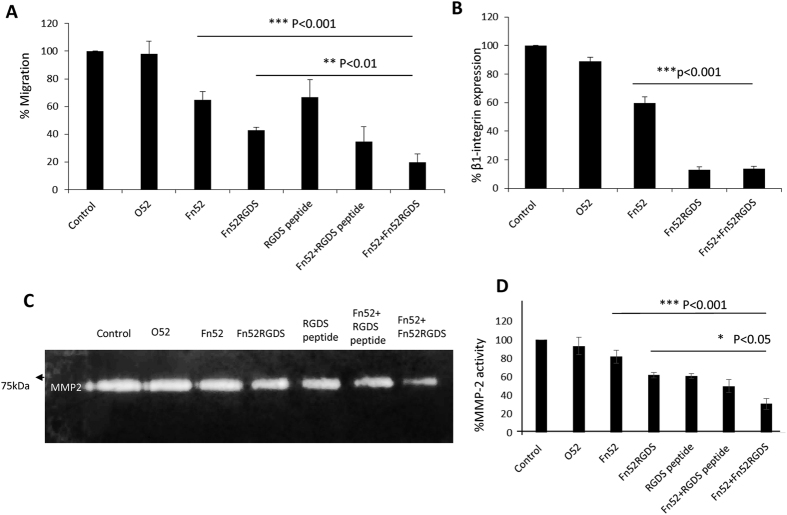
Effect of the scFv antibodies on migration, β1 integrin and MMP expression of lens epithelial cells. (Panel A) The extent of migration of lens epithelial cells was evaluated by transwell assay. Cells were grown in the presence of scFv antibodies and/or peptides, and TGF-β2. Control (no scFv) was assigned the value of 100%. (Panel B) Expression of β1 integrin was estimated by flow cytometry. Cells were treated with mouse anti-β1 integrin (1:400) and FITC-conjugated anti mouse antibody (1:200). MFI data was plotted as % fluorescence intensity. Three independent experiments were performed and mean ± SD was plotted; p less than 0.05 was taken as statistically significant. (Panel C) MMP activity was assessed by zymography. Culture supernatants obtained from the cultures of the migration assay were processed for zymography as described in the materials and methods section; control refers to cells without any scFv treatment. (Panel D) Densitometry corresponding to the zymograms (shown in Panel C) as analyzed by ImageJ software. Mean ± SD from two independent experiments was plotted.

Since migration of residual lens epithelial cells left over at the anterior lens capsule after cataract surgery, towards the posterior side of the lens capsule is an important contributor to the development of PCO, we evaluated the extent of migration of these cells with the scFv antibodies (Fig. 2). Significant reduction in migration of lens epithelial cells was observed in presence of the scFv antibodies directed against fibronectin, as compared to the controls (no scFv or irrelevant scFv O52) (Fig. 2A). Considering the migration of LECs to be 100% without any added scFv, our data shows that in the presence of scFv Fn52, the migration was 65% whereas it was 43% and ~20% in the presence of scFv Fn52RGDS and the combination (Fn52 + Fn52RGDS) of the antibodies respectively (Fig. 2A). Since the antibodies, scFv Fn52 and scFv Fn52RGDS differ by only an RGD sequence, these results suggest that the RGD motif present in scFv Fn52RGDS could have an influence on the migration of the LECs mediated through the blocking of cell surface integrins and interfering with fibronectin polymerization as well as cell-fibronectin interactions. In order to confirm this, we next evaluated the effect of RGD peptide alone and in combination with scFv Fn52 (to evaluate the synergism) on the migration of the lens epithelial cells (Fig. 2A). Addition of RGDS peptide to scFv Fn52 resulted in statistically significant reduction in migration (from 64.5% to 34.5%; p value < 0.001) akin to the reduction in migration observed when Fn52 was used in combination with Fn52RGDS (19.8%), signifying the importance of the RGD motif in mediating this effect.

Since it appeared that the RGD motif is effectively blocking the cell surface integrins, it was important to evaluate the expression of β1 integrin in the presence of the various scFv antibodies. The decrease in expression was quantified by flow cytometry (Fig. 2B), and the mean fluorescence intensity (MFI) was plotted, assuming the MFI of control set to be 100%. As expected, β1 integrin expression was significantly reduced; the extent of decrease was 40% in the presence of Fn52 and 87% in the presence of both, Fn52RGDS, as well as the combination of the two antibodies. The dramatic decrease seen in the presence of Fn52RGDS and the combination of the two antibodies also provides evidence for the binding of RGDS (in the scFv Fn52RGDS) to the integrins. In the presence of the scFv O52, decrease in β1 integrin was 11%, which was not statistically significant.

There is an increase in the levels of matrix metalloproteases following sham cataract surgery^12^; these MMPs facilitate cell migration by acting upon collagen and other members of the ECM^13^. We observed significant downregulation of levels of MMP-2 in the presence of Fn52RGDS, RGDS peptide, the combination of Fn52 and RGDS peptide and the combination of the two antibodies, when compared to the control where no scFv antibody was added or when the irrelevant antibody, O52 was used (Fig. 2C,D). MMP-2 expression was also reduced in the presence of scFv Fn52, but the decrease was not statistically significant (Fig. 2D).

### Assessment of FAK and SMAD signaling pathways

**Figure 3:**
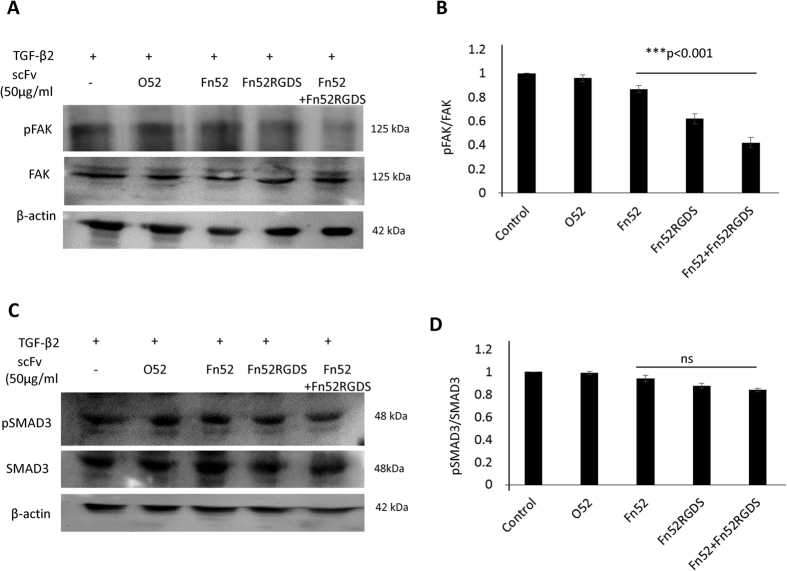
Assessment of FAK and SMAD signaling: (Panels A,C) Immunoblots showing the phosphorylation status of FAK and SMAD3 respectively. (Panels B,D) Bar diagrams represent the corresponding quantitative data.

Signal transduction pathways trigerred by integrins result in activation of focal adhesion kinase (FAK), causing regulation of cell proliferation and migration^14^. We therefore examined the phosphorylation status of FAK, and it was observed that in the case of Fn52RGDS as well as the combination of Fn52 and Fn52 RGDS, there was marked reduction in phosphorylation of FAK (Fig. 3A,B), with p value less than 0.001 in both cases.

Since SMAD-dependent signaling (canonical pathway) is commonly involved in response to TGF-β, the phosphorylation status of SMAD3 was also assessed, but it was not affected in any of the treatment groups (Fig. 3C,D).

### Assessment of the SMAD independent (non-canonical) signaling events mediated by the scFv antibodies

**Figure 4:**
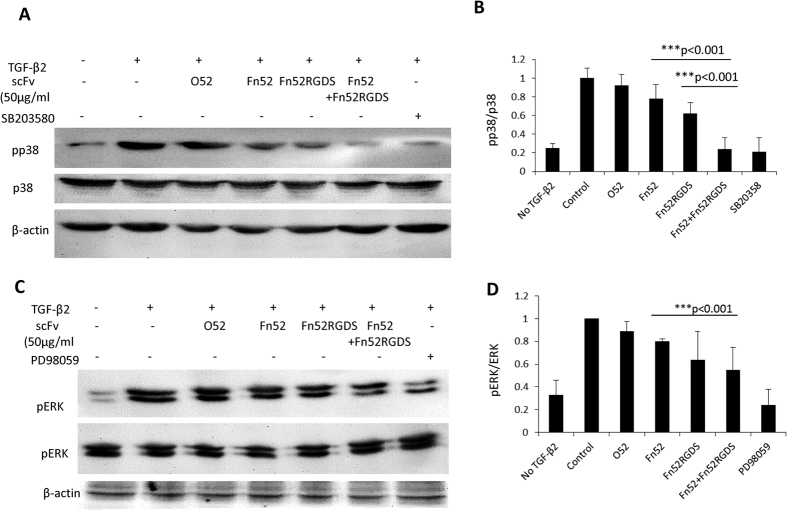
Evaluation of p38 and ERK signaling pathways in the presence of the antibodies: Primary LECs were allowed to reach 70% to 80% confluency, followed by serum starvation for 8–10 h, treatment with either scFv antibodies or inhibitors corresponding to p38 (SB203580) and ERK (PD98059) pathways for 1 h, followed by stimulation with or without TGF-β2 for 2 h. Phosphorylation status was assessed by western blotting. (Panel A) represents the immunoblot for phosphorylation status of p38 and (Panel B) shows the corresponding quantitation. (Panel C) represents the immunoblot for phosphorylation status of ERK and (Panel D) shows the corresponding quantitation for ERK.

In response to TGF-β, two pathways may be initiated, the SMAD-dependent (canonical) or the SMAD-independent (non-canonical). Since we had found no changes in the phosphorylation status of SMAD3, we investigated whether the MAP kinase signaling (SMAD-independent) pathway is involved in the action of scFv antibodies, because FAK is known to be involved in fibronectin-initiated signaling events to mitogen activated protein kinase (MAPK)^15^. The ratio of phosphorylated p38 to p38 was significantly reduced in the case of combination of the two antibodies (Fn52 and Fn52RGDS) when compared to either of the parent antibodies (Fig. 4A,B). The phosphorylation of ERK was also significantly reduced in the presence of Fn 52RGDS and the combination of the two scFv antibodies when compared to the parent antibody, scFv Fn52 (Fig. 4C,D).

### Changes in TGF-β2-induced EMT markers in the presence of scFv antibodies

**Figure 5:**
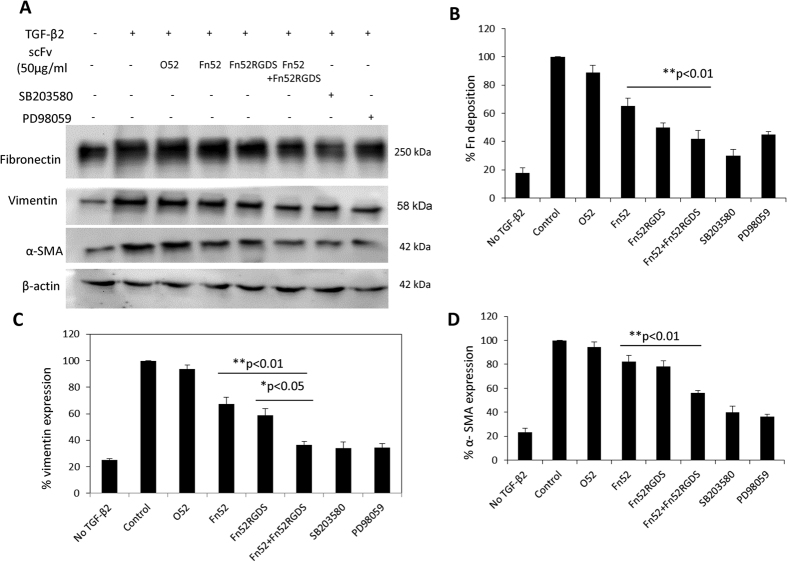
Assessment of EMT associated markers: Lens epithelial cells were grown in the presence/absence of scFv and presence or absence of TGF-β2. (Panel A) Western blot to analyze the EMT associated markers (insoluble cellular fibronectin (by DOC assay), vimentin and α-SMA) in the presence or absence of TGF-β2, scFv antibodies, and inhibitors for p38 and ERK pathways. (Panels B–D) Densitometry for the corresponding western blots shown in (Panel A), using ImageJ software. The figures represent mean ± SD derived from three independent experiments.

TGF-β2 is known to induce epithelial to mesenchymal transition (EMT) in any fibrotic pathology, with upregulation of EMT markers like fibrillar fibronectin, vimentin and α-SMA. We checked the basal levels of expression of the EMT markers in the absence of TGF-β, and in all cases, the expression of the markers was increased post-TGF-β treatment (Fig. 5). Treatment with scFv antibodies to the cultures of lens epithelial cells (induced with TGF-β), resulted in a significant decrease in the deposition of fibronectin and vimentin, and decreased levels of expression of α-SMA, as seen in the western blots in Fig. 5. Densitometry quantitation of the western blot showed that the extent of fibronectin deposition was significantly reduced in the presence of the scFv antibodies; assuming that the fibronectin deposition in the presence of TGF-β is 100%, the level of fibronectin was observed to be 18% in the absence of any TGF-β or scFv antibody, while it was 65% in the presence of Fn52, 50% in Fn52RGDS, and 42% in the presence of the combination of antibodies (Fig. 5B). scFv O52 resulted in non-significant change in fibronectin deposition. Quantitation of western blot signals for vimentin showed a similar trend as fibronectin (Fig. 5C). The expression of vimentin in the absence of TGF-β or scFv was 25%, and in the presence of TGF-β and the various antibodies, it was as follows: Fn52 (67%), Fn52RGDS (58%) and combination (36%). It may be noted that vimentin expression was significantly lowered in the presence of the combination of the two antibodies as compared to the individual parent antibodies. There was no significant change in the level of expression of α-SMA in the presence of the scFv Fn52 (82%), while it was significantly decreased in the presence of Fn52RGDS (78%) and the combination of the two antibodies (56%), as compared to the control (Fig. 5D). In agreement with previous studies^16^,^17^, inhibitors for ERK and p38 signaling pathways resulted in significant decrease in expression of the EMT markers (Fig. 5).

### Collagen gel contraction

**Figure 6:**
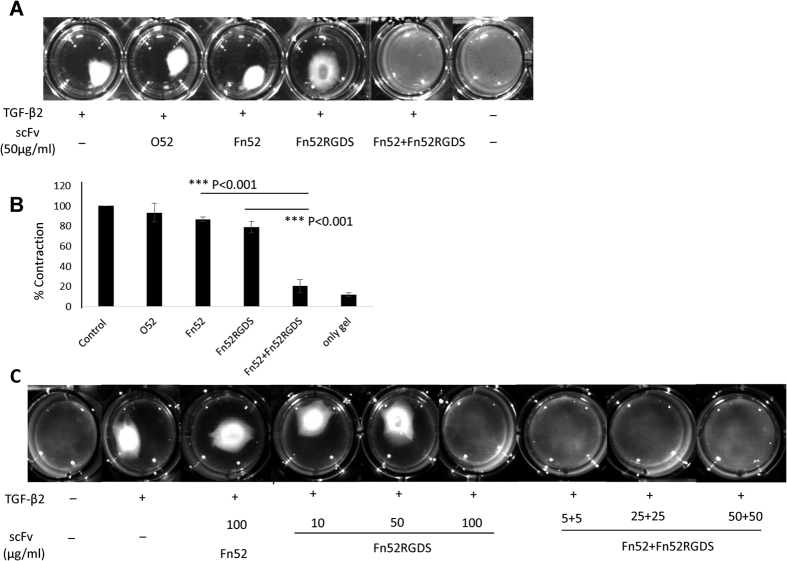
Evaluation of efficacy of antibodies in inhibiting collagen gel contraction: Collagen gel contraction was performed using lens epithelial cells in the absence (control) or presence of the scFv antibodies along with TGF-β2. Control culture included cells mixed with collagen and allowed to solidify, with no TGF-β2 added. Contraction was recorded after 24 hours of overlaying with serum-free media. (Panel A) scFv antibodies used at concentration of 50 μg/ml; (Panel B) Area of the collagen gel (for Panel A) was measured using Image J software and plotted as percent contraction. Contraction in the control set was assigned a value of 100%. (Panel C) Collagen gel contraction performed at varying concentrations of scFv antibodies.

It has been reported by Wormstone *et al*. that MMP-2 plays a crucial role in the contraction and wrinkling of the lens capsule^18^. In light of this, since MMP-2 expression was found to be decreased in the presence of the scFv antibodies, it was expected that these antibodies would also inhibit collagen gel contraction (an assay used to mimic the lens capsule contraction). Figure 6A shows the images of the collagen gel contraction assay carried out at a concentration of 50 μg/ml; panel B shows the quantitative data corresponding to Panel A; in the presence of Fn52, the contraction was comparable to that of the control (no scFv) and the irrelevant antibody (O52). Surprisingly, the contraction was almost abolished in the presence of the combination of antibodies (Fn52 + Fn52RGDS, using a cocktail of 25 μg/ml each).

In order to investigate whether there was a quantitative (dose-dependent) effect of the two antibodies, we carried out the same experiment at varying concentrations of Fn52RGDS, and the combination of Fn52 and Fn52RGDS. As seen from Fig. 6C, gel contraction was maximum in the presence of TGF-β2; no inhibition was achieved with Fn52 at a concentration of 100 μg/ml; however, 100 μg/ml of Fn52RGDS was able to achieve complete inhibition of collagen gel contraction. The same extent of inhibition was achieved with very low dose of the cocktail (starting from a total concentration of only 10 μg/ml, i.e., 5 μg/ml of each of Fn52 and Fn52RGDS). In comparison, 10 μg/ml of Fn52RGDS alone is clearly not enough to inhibit collagen gel contraction; inhibition with this antibody requires a concentration of 100 μg/ml. Clearly, therefore, the two antibodies act in a synergistic manner to effect the inhibition of collagen gel contraction.

### Evaluation of extent of diffusion of the scFv antibodies in the ECM

**Figure 7:**
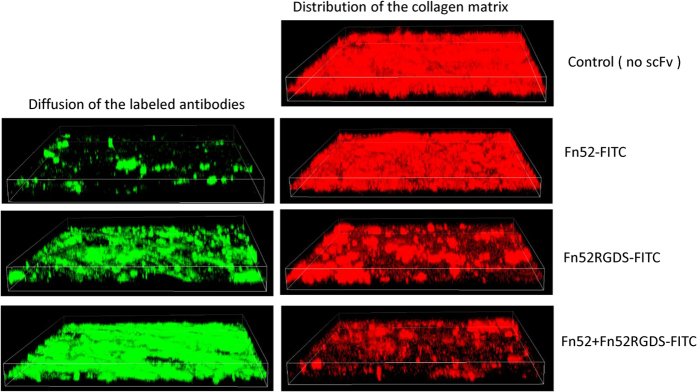
Assessment of level of diffusion and collagen deposition. The left panel shows the diffusion of scFv antibodies alone or in combination, through pre-formed ECM, assessed by evaluating the distribution of scFv antibodies (labeled with FITC) in the 3D matrix using confocal microscope. The right panel shows the extent of freshly laid collagen (using anti-collagen type-I antibody, probed with PE-labeled anti-rabbit antibody) in the time period after addition of scFv antibodies till termination of the assay (10 days). The images are 3D renderings of z-stack confocal images.

We next evaluated the diffusion of the scFv antibodies through a pre-formed ECM. It is evident (Fig. 7) that the distribution and diffusion of the scFv antibodies is maximal and uniform throughout the matrix when the antibodies were used in combination, which is not the case when the individual antibodies were used. scFv Fn52 diffused minimally, followed by scFv Fn52RGDS, and maximum diffusion was observed when both the antibodies were used in combination (scFv Fn52RGDS-FITC) (Fig. 7).

### Evaluation of extent of collagen deposition

We assessed the synthesis/deposition of collagen, mediated by TGF-β (by the LECs), post addition of the scFv antibodies in the transwells used for diffusion studies (Fig. 7). Clearly, collagen synthesis was dramatically reduced in the presence of the cocktail of antibodies.

## Discussion

Post cataract surgery, the abundance of cytokines and growth factors in the aqueous humor promotes lens epithelial cell proliferation and migration, along with exaggerated synthesis and deposition of extracellular matrix. Among the cytokines, TGF-β2 plays a predominant role, and fibronectin and collagen form the main components of the scaffold of the ECM, providing anchorage for the LECs. The polymerization of meshwork of fibronectin aids in cell survival, proliferation, adhesion and migration, all of which are fundamental features of any fibrotic disease.

Fibronectin is an important regulator of cell activities through its direct interaction with the cell surface integrin receptors, which leads to the formation of adhesion complexes and engagement with the actin cytoskeleton. The signals generated from this interaction combined with those arising from the action of various cytokines and growth factors (such as TGF-β2)^19^, facilitate the self-polymerization of Fn, as well as regulate the formation as well as maintenance of cell–matrix fibrillar adhesions^20^. It is not surprising therefore, that fibronectin plays a critical role in mediating ECM deposition and inflammatory response in the development of fibrotic diseases. *In vitro* experiments with rabbit lens epithelial cells have demonstrated the role of fibronectin in promoting the adhesion and migration of these cells^10^,^21^, which contribute to the fibrotic pathology in posterior capsular opacification, through their differentiation into myofibroblasts in the presence of TGF-β2. Although fibronectin is present in minimal amounts in adult lens, exposure to plasma fibronectin and other growth factors, results in increased expression of fibronectin, following a cataract surgery^22^. An examination of sections of the anterior capsules obtained from cataract surgeries, highlighted the importance of integrins in the adhesion of the lens epithelial cells to the lens capsule, which could be important in the cell-posterior capsule interaction after cataract surgery^23^.

Given the immense importance of fibronectin in the causation of PCO, we expected that inhibition of both, the fibronectin polymerization as well as Fn-cell surface integrin interactions, may be useful in inhibition of fibrotic changes seen in the TGF-β2-induced lens epithelial cells. Previously we have explored the effect of such inhibition in the context of another ocular fibrotic process (in proliferative vitreoretinopathy or PVR), using single chain variable fragment antibodies targeted to the N-terminal 30 kDa region of fibronectin (Fn52) or its re-engineered form (Fn52 RGDS)^11^.

The amino terminal domain of fibronectin, spanning the first five type I repeats (I1-5), constitutes a major site for Fn self-association^8^,^24^,^25^,^26^, playing a critical role in Fn fibrillogenesis. The scFv Fn52 binds to a cryptic epitope within this region, and has been shown to be important in the fibronectin polymerization process, while Fn52 RGDS was shown to possess dual binding properties - to the N-terminal region of fibronectin like its parent version, as well as to the cell surface integrins. Thus Fn 52RGDS not only inhibits fibronectin polymerization, but also blocks fibronectin-cell surface interactions^11^.

Although as just described above, the individual scFv antibodies have been previously shown to have the desired anti-fibrotic effects, the present study is novel in that it emphasizes the fact that using these antibodies as a cocktail is more beneficial; our data shows that this may be attributed to the increased ability of the antibodies to diffuse through a pre-formed matrix. Below, we summarize the features in respect of which the synergism of the two antibodies is evident and therefore suitable for targeting a fibrotic disorder such as PCO.

(i) Actin-stress fiber arrangement was drastically altered in the cultures treated with a cocktail of the two antibodies, with reduced actin-stress fiber formation which was mainly localized towards the periphery; (ii) The cocktail of antibodies significantly reduced the migration of LECs as compared to scFv Fn52 alone (p value, 0.001) or scFv Fn52RGDS (p value 0.01); (iii) The expression of MMP-2 likewise also showed a significant reduction when the combination of antibodies was used, as opposed to independent antibodies (Fn52: p value < 0.001; Fn52RGDS: p value < 0.05); (iv) Further, our results indicate that the combination of both the antibodies almost abolished the TGF-β-induced contraction of collagen; at a dose of 100 ug/ml, Fn52 alone was not able to inhibit collagen gel contraction, while Fn52RGDS inhibited the contraction at a concentration of 100 ug/ml. Intriguingly, the same extent of inhibition was seen with the cocktail of the two antibodies used at a concentration as low as 5 μg/ml each, clearly suggesting that the two work in synergism to show the desired effect; (v) The synergism of the two antibodies is also displayed in the increased ability to diffuse through a pre-formed matrix as well as a reduced tendency to lay fresh ECM component such as collagen.

Considering the above, the data suggests that the combination of these antibodies possibly operates through the integrin-mediated stimulation of focal adhesion kinase, which plays a key role in controlling cell migration and contraction.

Ligand binding to integrins causes FAK to transduce signals to mitogen activated protein kinase (MAPK)^15^. Since the scFv antibodies were effective in lowering the expression of β1 integrin, the effect on phosphorylation status of ERK1/2 and p38 was evaluated. Phosphorylation of ERK1/2 was not affected by the presence of the scFv Fn52, but Fn52RGDS and the combination of the two antibodies caused marked reduction in phosphorylation (p < 0.001). The phosphorylation of p38 was significantly reduced in the presence of scFv Fn52, Fn52RGDS as well as the combination of the antibodies (p < 0.001). Thus the action of these antibodies is through inhibition of the non-canonical pathway in response to TGF-β, along the integrin-dependent focal adhesion kinase (FAK) activation pathway^27^ and through the MAP kinase pathway^28^. This also explains the results obtained with respect to integrin and fibronectin expression^27^, actin stress fiber alteration, fibronectin matrix organization and cell migration, since previous work has shown a functional role of FAK in TGF-β-mediated EMT^29^.

**Figure 8:**
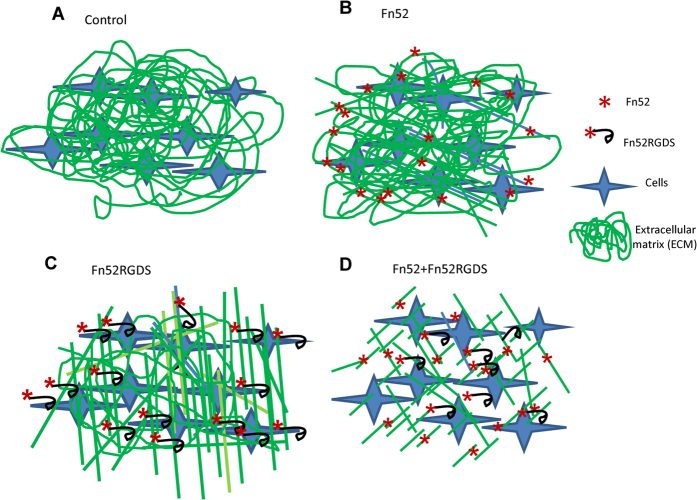
Schematic diagram proposing a possible explanation for the synergism seen in the presence of the cocktail of Fn52 and Fn52RGDS. (**A**) represents the dense meshwork of ECM proteins that exists in the context of fibrosis; (**B**) scFv Fn52 binds to the 30 kDa N-terminal region of fibronectin, and inhibits its polymerization; (**C**) Fn52RGDS similarly also binds to the N-terminal region of fibronectin. In addition, it is capable of binding to the cell surface integrin receptors, and inhibits not only fibronectin assembly, but also Fn-cell interactions; (**D**) In cultures treated with the cocktail of antibodies, scFv Fn52 is proposed to act by inhibiting fibronectin polymerization, leading to a less dense ECM, thereby facilitating the diffusion of the other scFv, Fn52RGDS throughout the ECM. The co-operative action (or synergism) of the scFv antibodies could result in an increased inhibitory action of the cocktail.

It has been shown that in many tumors, the dense matrix of collagen (and other ECM components) inhibits therapeutic drug penetration, diffusion and transport, acting as a barrier in drug delivery. Therefore, for tumors with a well developed collagen network, treatments that reverse or inhibit collagen production and assembly have been proposed in order to achieve better drug delivery^30^. In the case of TGF-β2-mediated EMT of the LECs, the extensive synthesis of fibronectin, and its binding to ligands such as α5β1 receptor, is expected to regulate collagen fibril structure^31^. The resulting increase in meshwork of fibronectin and collagen will likewise result in lowering of the availability of the scFv molecules (or any other drugs) for action on the cells. In light of this, the diffusion study clearly highlights the potential of the cocktail to provide for increased availability of the antibodies through the dense meshwork of the ECM assessed using fluorescently-tagged antibodies. Based on these observations, we rationalize that it is possible that the interaction of scFv Fn52 with any available soluble fibronectin, causes stretching and exposure of cryptic binding sites in fibronectin, and thereby inhibits fibronectin polymerization, which in turn leads to a loosening of the matrix, aiding in better diffusion of Fn52RGDS throughout the ECM. The diffused scFv Fn52RGDS now has the freedom to bind to the cell surface integrin as well as to the immature fibrils (provided by the action of Fn52). The action of Fn52RGDS in turn, further helps in the diffusion of Fn52 (illustrated by a schematic in Fig. 8). In this way a co-operative action of the two scFvs could be envisaged to result in more effective inhibitory action of the cocktail of antibodies.

An important point to consider is why the combination of antibodies is at all necessary, i.e., why is Fn52RGDS alone not sufficient to achieve the desired effects? A possible explanation might stem from the different natures of the two functions performed. The RGD sequence causes antibodies carrying this sequence to become titrated to locations on cell surfaces carrying integrin molecules. Since the numbers of integrin molecules on the surfaces of all cells present could be such as to titrate away the bulk of the Fn52RGDS population with a kinetics faster than would allow the same antibody to significantly inhibit fibronectin polymerization all over the extracellular milieu, it might be necessary to either supply more Fn52RGDS than is titratable by the integrin, or to supply something else to perform the inhibition of fibronectin polymerization all over the extracellular milieu (and away from the cell surface). Therefore, the Fn52 antibody is necessary to be additionally present, in numbers sufficient to block fibronectin polymerization. This would suggest that what is required is ideally a combination of Fn52 (which acts insufficiently, when it is acting alone) and any reagent carrying the RGD sequence, e.g., reagents like eptifibatide, and not necessarily the Fn52RGDS reagent. However, it is necessary to consider here that there is also an especial need to inhibit fibronectin polymerization extremely locally, in the immediate vicinity of the cell surface – and if one were to use eptifibatide and Fn52, one would need to use much higher concentrations of Fn52 to ensure sufficient inhibition of fibronectin polymerization occurring immediately next to the cell surface. This need could be substantially reduced through the use of a bi-functional reagent which both binds to integrins and also inhibits fibronectin polymerization locally, in the immediate molecular vicinity of the cell surface, so that there is a gap maintained between the cells and the forming (nascent) extracellular matrix, which prevents cells from integrating completely into an unyielding extracellular matrix. Thus, the ideal combination of reagents is one that has a component which inhibits fibronectin polymerization everywhere, and a second component that ensures both (a) that fibronectin polymerization is inhibited locally in the vicinity of the cell surface (in this case, without relying on the availability of diffusible Fn52 molecules which could be acting in the entire milieu) and (b) that integrins are blocked at their RGD binding sites, preventing their interactions with fibronectin.

The ECM clearly acts as a limiting factor in the diffusion of drugs to the site of action, explaining the reduced efficacy of drugs when these are used in the context of diseases where extensive synthesis and deposition of ECM are involved. In such cases, it would be beneficial to design combination therapies, where one component facilitates the diffusion of the other component and vice versa to achieve maximal results at minimal concentration.

## Methods

### Ethics Statement

Informed consent for use of tissue (anterior capsule) was obtained from all participants enrolled in the study. Institutional Review Board of the institute (PGIMER) accorded approval of the study protocol (NKG/707). The guidelines of the Declaration of Helsinki were observed.

### Cell culture

Primary culture of lens epithelial cells was set up as described previously^32^. Briefly, lens capsules derived from patients undergoing cataract surgery were collected in sterile vials containing media (MEM with 50 ug/ml of gentamycin), and plated on tissue culture plate (24 well format) in the presence of complete media (MEM + 20%FBS along with 50 ug/ml of gentamycin). Media was replenished after every 3–4 days and cells were harvested at 60–70% confluency and further propagated. For all experiments, cells were used after the 3rd passage.

At the time of experiment, lens epithelial cells were seeded and grown till 80–90% confluency in complete growth media, followed by serum starvation for 6–8 h. Cells were treated with scFv antibodies Fn52, Fn52RGDS and O52 alone or in combination (Fn52 and Fn52RGDS) along with TGF-β2 (2 ng/ml) in serum-free media, and incubated for the indicated time periods.

### Western Blotting

Lens epithelial cells were grown in the presence of serum-containing media for 24 h, followed by serum starvation and treatment with scFv antibodies and TGF-β2 (2 ng/ml) for 24 h. Cells were washed and lysed; cell lysate was boiled in 1X SDS-PAGE sample loading buffer, and proteins were separated on 10% SDS-PAGE. The separated proteins were transferred onto the nitrocellulose membrane and blocked with 10% skimmed milk overnight at 4 °C. The membrane was probed with the primary antibody, followed by secondary HRP-conjugated antibody. The signal was visualized by ECL reagent and captured on the FluorChem E Imaging System (Protein Simple).

### Immunofluorescence

Primary culture of lens epithelial cells were grown in complete media (MEM + 20%FBS) for 24 h; cells were serum starved for 8–10 h, and treated with scFv antibodies in the presence of TGF-β2 (2 ng/ml) for 24 h. Cells were fixed with 4% paraformaldehyde, permeabilized using 0.5% triton-100X and 0.05% tween-20, and blocking was done with 5% BSA. Following treatment with primary and secondary antibodies, images were acquired using Olympus confocal microscope.

### Evaluation of viability and proliferation

Primary culture of lens epithelial cells were grown at a density of 10,000 cells/well in a 96-well plate in MEM + 20% FBS. Cells were serum starved followed by addition of the scFv antibodies Fn52, Fn52RGDS and a combination of both Fn52 + Fn52RGDS along with TGF-β2 (2 ng/ml), in dose-dependent manner and incubated for 24 h. For evaluating the toxicity of scFv, at the end of the incubation, MTT (3-(4,5-dimethylthiazol-2-yl)-2,5-diphenyltetrazoliumbromide); stock, 5 mg/ml) was added to a final concentration of 10% of the total volume in the well, and incubated for 3–4 h to allow for formazan crystal formation. Controls were likewise set up to evaluate the effect of PBS on cells. The formazan crystals were dissolved in 100 μl of DMSO per well and optical density was read at 570 nm with reference at 630 nm.

Proliferation was evaluated by estimating BrdU intake. Cells were seeded as mentioned above in the presence and absence of scFv antibody in a dose dependent manner, followed by addition of BrdU (10 uM per well). After 6–8 h, media was removed and fixation of the cells was done with methanol/H_2_O_2_ for 1 h. Denaturation was done using 2N HCl for 30 min followed by blocking using 5% BSA in PBS. Incorporation of BrdU was assessed by using anti-BrdU antibody raised in mouse (1:1000) for 1 h, followed by incubation with anti-mouse HRP-conjugated secondary antibody (1:5000) for 1 h. Color was developed with TMB and was quantified by reading at 450 nm. Another scFv, O52 was used as a negative control.

### Transwell Migration assay

Migration of the lens epithelial cells was assessed by transwell assay. Briefly, lens epithelial cells were harvested and pre-incubated with TGF-β2 (2 ng/ml) and the following reagents for 1 h: (i) scFv O52 (50 μg/ml; negative control), (ii) scFv Fn52 (50 μg/ml), (iii) scFv Fn52 RGDS (50 μg/ml), (iv) RGDS peptide (5 μg/ml), (v) scFv Fn52 (50 μg/ml) + RGDS peptide (5 μg/ml) (vi) scFv Fn52 + scFv Fn52RGDS (25 μg/ml each). Cells were seeded on the filter in the top chamber of the trans-well plate (8.0-μm pore; Corning^®^ Costar^®^ Transwell^®^ cell culture inserts) in a 24-well format, in a total volume of 300 μl. The lower chamber contained the chemoattractant (fibronectin, 0.02 μM, purified from human plasma). The plates were incubated for 8 h at 37 °C and 5% CO_2_. Migration of the cells was evaluated by staining the cells migrated on the lower surface of the filter using crystal violet stain and OD 560 of the eluate. All experiments were performed four times.

### MMP expression

The level of expression of MMP2 was evaluated using the culture supernatant (obtained from the cell migration experiment set-up described above) by gelatin zymography^33^. Briefly, gelatin (1 mg/ml) was co-polymerized in the lower separating gel of a 10% SDS-PAGE and samples for electrophoresis were prepared with non-reducing sample loading buffer. SDS was removed by incubation with 2.5% triton-X100 for 1 h, followed by incubation in activity buffer overnight at 37 °C. Gel was stained with Coomassie blue dye and then de-stained to visualize the zone of clearance.

### Examination of extent of diffusion of antibodies through a pre-formed ECM

For the diffusion assay, scFv antibodies were conjugated with FITC according to the manufacturer protocol (Pierce™ FITC Antibody Labeling Kit). Lens epithelial cells were seeded onto the transwell (0.4 μm pore size; 12 well format) pre-coated with collagen type I, in complete media (MEM plus 10% FBS). Once a monolayer was formed, media was replaced with serum free media supplemented with TGF-β2 (2 ng/ml), and allowed to grow for a period of 3–4 weeks, for synthesis of extracellular matrix. At the end of the incubation, FITC labeled scFv antibodies were added in the following combinations: (i) scFv Fn52-FITC, (ii) scFv Fn52RGDS-FITC, and (iii) scFv Fn52 + scFv Fn52RGDS-FITC, and were allowed to diffuse through the pre-formed matrix over a period of 8–10 days. TGF-β2 was replenished periodically at an interval of 3 days throughout the course of the experiment, while labelled antibodies were replenished at an interval of 3 days for the 8–10 day period. The extent of diffusion was analyzed by observing the fluorescence of labelled scFv antibodies in a 3D view by confocal microscopy.

### Assessment of fibronectin polymerization and actin-stress fiber rearrangement

For evaluating the effect of scFv antibodies on fibronectin polymerization and actin-stress fiber rearrangement, lens epithelial cells were seeded on glass cover slips in the presence of complete media and incubated overnight, followed by serum starvation and treatment with scFv antibodies in the presence of 2 ng/ml TGF-β2for 24 h. After fixation, permeabilization and blocking, cells were incubated with primary antibody (anti-fibronectin antibody raised in rabbit), followed by secondary antibody (anti-rabbit-FITC conjugate). For studying the actin-stress fiber rearrangement, the same slides were treated with Phalloidin-TRITC conjugate, and counter-stained with DAPI (nuclear stain).

### Evaluation of the EMT specific markers

The deposition of insoluble fibronectin was assessed by DOC (Deoxycholate) insolubility assay as described previously^11^. 1 × 10^6^ cells were plated on a 6 well plate in the presence of serum-containing media, followed by serum starvation for 7–10 h. Cells were either treated with inhibitors (10 μM) of p38 and ERK pathways or with the relevant scFv antibodies (with 2 ng/ml TGF-β2). Cells with TGF-β2 alone, were treated as controls. To evaluate the basal level of expression of EMT markers, cells were also grown in the absence of TGF-β2 or any added scFv antibody. After 24 h, DOC lysis buffer was used for cell lysis^11^. The cell suspension was centrifuged at 14,000 rpm for 1 h to separate the DOC soluble and insoluble fractions. The DOC insoluble fraction was separated on 7.5% SDS PAGE under reducing conditions, followed by transfer of proteins onto a nitrocellulose membrane and immunoblotting with anti-fibronectin antibody (Sigma Chemicals, 1:5000). Chemiluminescence imaging was carried out (FluorChem E imaging system), and Image-J software was used for quantitation. Anti-β actin antibody (Sigma Chemicals; 1:5000) was used as a loading control in the DOC-soluble fraction.

For the assessment of vimentin and α-SMA expression, western blotting was done. The soluble fraction obtained after the DOC assay was used for western blotting. Cell lysate was boiled in 1X SDS-PAGE sample loading buffer and proteins were separated on 10% SDS-PAGE. The separated proteins were transferred onto the nitrocellulose membrane and blocked with 10% skimmed milk overnight at 4 °C. The membrane was probed with primary anti-vimentin and anti-α-SMA antibodies raised in mouse, followed by the secondary anti-mouse HRP conjugated antibody. The blot was detected using ECL reagent and image was acquired on FluorChem E imaging system.

Flow cytometry was used to evaluate the expression of β1 integrin expression. Lens epithelial cells were seeded and treated in a similar fashion as described for the DOC assay. At the end of the incubation, cells were detached and blocked in 2% BSA-PBS, after which all the steps were carried out on ice. Post blocking, cells were treated with primary anti-β1 integrin antibody raised in mouse, followed by anti-mouse-FITC conjugated secondary antibody. BD FACS-Aria system was used to record the mean fluorescent intensity; MFI of the control was set to 100%.

### Collagen gel contraction

Wells of a 24 well plate were coated with 400 μl of BSA-PBS (5 mg/ml) overnight at 37 °C and BSA was removed just before performing the collagen gel contraction. Cells were trypsinized and washed twice with serum free media. 25,000 cells were incubated with scFv antibodies for 1 h followed by mixing with neutralized collagen solution. The cell-collagen mix with TGF-β2 (2 ng/ml) was added to the wells pre-coated with BSA, and allowed to solidify for 90 min. In the positive control, only cell-collagen mix along with TGF-β2 was added, while in the negative control, only cells were added in the collagen mix. At the end of 90 min, 600 μl of serum free media was overlaid on the collagen gels with force, so that the gel floats in the serum free media. Contraction of the gel was monitored and the area was calculated using image-J software. Contraction in the control set was taken to be 100%.

### Assessment of cell-signaling pathways

5 × 10^5^ lens epithelial cells were grown in a 6-well plate in the presence of serum-containing media, followed by serum starvation for 6–8 h. Post starvation, cells were treated with inhibitors for p38 and ERK pathways (10 μM) or with scFv antibodies and TGF-β2 (2 ng/ml) was added 1 h after the addition of the reagents, and was further incubated for 2 h. At the end of the incubation, cells were washed twice with PBS, harvested and lysed in 100 μl of ice cold RIPA buffer (25 mM Tris-HCl [pH 7.6], 1 mM Na_3_VO_4_, 1% sodium deoxycholate, 150 mM NaCl, 0.1% SDS, 1% NP-40) with a cocktail of protease inhibitors. Cell lysate was separated on 10% SDS-PAGE, transferred on the nitrocellulose membrane, blocked with 2% BSA for 1 h and probed with primary anti-pFAK (Y397, 1:500; Sigma Chemicals), anti-pSMAD3 (Y208; Sigma Chemicals), anti-pERK (Th202/Tyr204; Cell Signaling), or pp38 (Th180/Tyr182; Cell Signaling) antibodies, followed by anti-rabbit-HRP conjugate antibody (1:10,000). The membrane was developed using ECL reagent (Bio-Rad) and acquired on FluorChem E Imaging System. For evaluation of the corresponding total protein levels of each molecule, stripping and re-probing of the membrane was carried out with anti-FAK, Anti-SMAD3, anti-ERK and anti-p38 antibodies. Equal loading was ensured by probing with anti-β-actin antibody.

### Statistical Analysis

Results are expressed as mean ± SD. One-way Anova test was used for analysis of data. A p value < 0.05 was considered to be significant.

## Additional Information

**How to cite this article**: Tiwari, A. *et al*. Control of fibrotic changes through the synergistic effects of anti-fibronectin antibody and an RGDS-tagged form of the same antibody. *Sci. Rep*. **6**, 30872; doi: 10.1038/srep30872 (2016).

## Supplementary Material

Supplementary Information
