# Supplementary material for: Control of fibrotic changes through the synergistic effects of anti-fibronectin antibody and an RGDS-tagged form of the same antibody
Source: Sci Rep. 2016 Aug 3;6:30872. doi: 10.1038/srep30872 (PMC4971484; doi:10.1038/srep30872)
Supplement: Supplementary Information [file srep30872-s1.pdf]

Supplementary material for the manuscript:

Title: Control of fibrotic changes through the synergistic effects of anti-fibronectin antibody and an RGDS-tagged form of the same antibody

Authors: Anil Tiwari, Rajendra Kumar, Jagat Ram, Maryada Sharma and Manni Luthra-Guptasarma

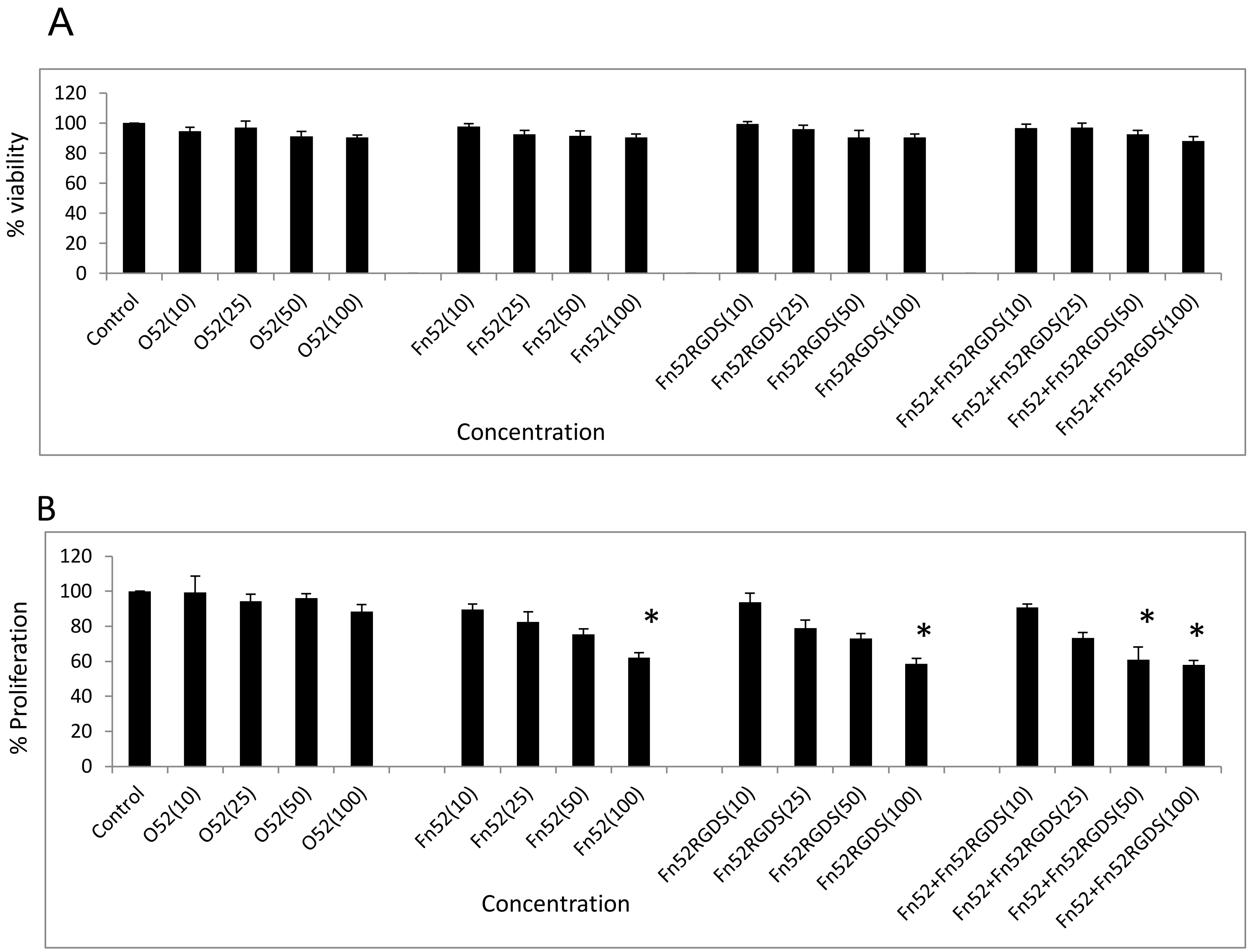

Supplementary Fig 1: Effect of scFv Fn52 (50µg/mL), Fn52RGDS (50µg/mL) and combination of scFv antibodies (Fn52+Fn52RGDS; 25+25 µg/mL) on viability (Panel A) and proliferation (Panel B) of lens epithelial cells. Panel A: Viability of the lens epithelial cells was evaluated by MTT assay. Control (assigned as 100%) indicates the condition where lens epithelial cells were seeded along with TGF-β2 (2ng/ml) in the absence of any scFv antibody. Panel B: Proliferation of the lens epithelial cells was assessed by incorporation of BrdU. The optical density of the control (absence of scFv) was taken as 100%. scFv O52 was used as an irrelevant (negative) control. $p \leq 0.5$ was considered statistically significant.
